# Increasing impact of urban fine particles (PM_2.5_) on areas surrounding Chinese cities

**DOI:** 10.1038/srep12467

**Published:** 2015-07-29

**Authors:** Lijian Han, Weiqi Zhou, Weifeng Li

**Affiliations:** 1 State Key Laboratory of Urban and Regional Ecology, Research Center for Eco-Environmental Sciences, Chinese Academy of Sciences, Beijing 100085, China

## Abstract

The negative impacts of rapid urbanization in developing countries have led to a deterioration in urban air quality, which brings increasing negative impact to its surrounding areas (e.g. in China). However, to date there has been rare quantitative estimation of the urban air pollution to its surrounding areas in China.We thus evaluated the impact of air pollution on the surrounding environment under rapid urbanization in Chinese prefectures during 1999 – 2011. We found that: (1) the urban environment generated increasing negative impact on the surrounding areas, and the PM_2.5_ concentration difference between urban and rural areas was particularly high in large cities. (2) Nearly half of the Chinese prefectures (156 out of 350) showed increased impact of urban PM_2.5_ pollution on its surrounding areas. Those prefectures were mainly located along two belts: one from northeast China to Sichuan province, the other from Shanghai to Guangxi province. Our study demonstrates the deterioration in urban air quality and its potential impacts on its surrounding areas in China. We hope that the results presented here will encourage different approaches to urbanization to mitigate the negative impact caused by urban air pollution, both in China and other rapidly developing countries.

China’s rapid urban and economic development over a short period has not only led to better living standard, but also caused severe environmental pollution, particularly air pollution, in urbanized regions^1^,^2^,^3^. Despite the decrease in “traditional pollutants” (e.g. NO_2_, SO_2_), fine particulate matter (PM_2.5_) has became a major air pollutant that threatens human health, including morbidity and mortality, and decreases meteorological visibility^4^,^5^. As this major urban air pollutant increases both totally and proportionally in Chinese cities, concentrations of PM_2.5_ have attracted increasing concern due to its effects on visibility and public health^3^,^6^. Owing to the differences in emission magnitudes, urban and rural areas exhibit heterogeneous PM_2.5_ concentrations, which indicate varied interactions between urban and surrounding areas^3^. When PM_2.5_ concentrations are higher in urban areas than surrounding areas, urban air pollution can negatively impact the surrounding rural areas; when PM_2.5_ concentrations are lower in urban areas than surrounding areas, urban air quality can be negatively impacted by the surrounding rural areas. To monitoring PM_2.5_ concentration changes, the monitoring networks have been well established in many developed countries, but fewer in developing countries which were suffering severe PM_2.5_ pollution^7^,^8^. Critically, networks with limited spatial distribution make it difficult to quantitatively illustrate the spatial patterns and impacts of urban air pollution on the surrounding rural areas. Thus, remote sensing derived PM_2.5_ concentrations were introduced for large-scale air quality analysis.

Remote sensing and modeling derived PM_2.5_ concentration records suggest that PM_2.5_ concentrations are higher in many regions of China than in other countries, particularly in urban areas^9^,^10^. Few studies, however, have quantitatively examined the annual or multi-year averaged spatial pattern of PM_2.5_ concentrations in Chinese cities, or the impact of urbanization on PM_2.5_ concentrations^11^, and multi-year analysis is limited due to poor long-term large-scale PM_2.5_ concentration data^3^,^12^. However, this information is critically important for China to achieve the recently released long-term plan for controlling air pollution (http://www.gov.cn/zwgk/2013-09/12/content_2486773.htm) and to accomplish the new-type urbanization plan. Therefore, the objectives of this study were to examine the changes in PM_2.5_ concentration differences between urban and surrounding areas, and suggest better air quality controls to policy makers in China.

## Results

**Figure 1:**
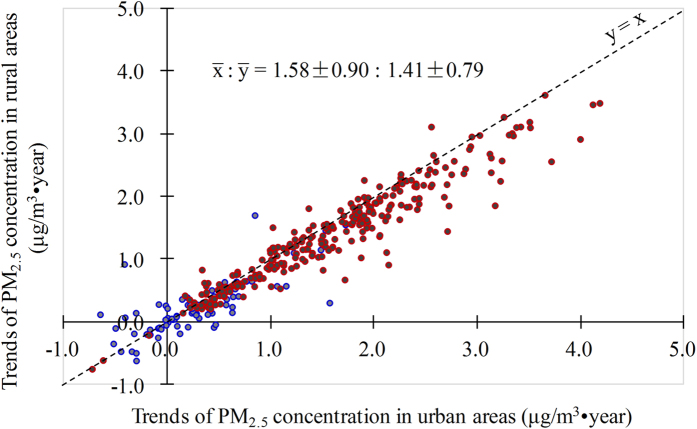
Increasing trends of PM_2.5_ concentrations in urban areas (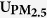) compared with those in rural areas (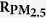) at Chinese prefectures from 1999 to 2011. Red dots indicate cities with significant PM_2.5_ concentration trends in both urban and rural areas, blue dots represent cities without significant PM_2.5_ concentration trends in either urban or rural areas.

**Figure 2:**
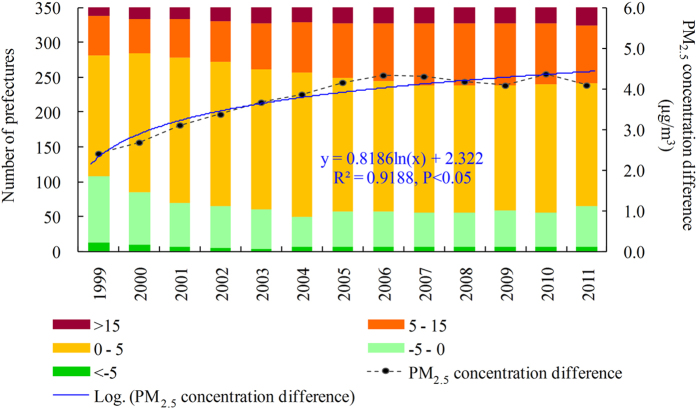
Mean PM_2.5_ concentration difference (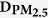) between urban and rural areas in Chinese prefectures from 1999 to 2011. The bars represent number of cities with each PM_2.5_ concentration difference group, and the dots represent prefectural mean PM_2.5_ concentration difference (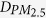) in China during 1999–2011.

PM_2.5_ concentration in the urban areas (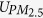) showed a stronger increase trend compared with the trend of PM_2.5_ concentration in the urban areas (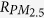) (Fig. 1; 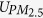 trends : 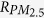 trends = 1.58 ± 0.90 : 1.41 ± 0.79), indicating a stronger impact of human activities in urban areas than in rural areas of China. The stronger increases of 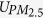 resulted in significantly increased PM_2.5_ concentration difference (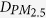) (Fig. 2; R^2^ = 0.9188, P < 0.05). The prefectural averaged 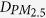 was 2.41 μg/m^3^ in 1999, but was increased to 4.09 μg/m^3^ in 2011. The amount of prefectures with 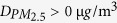 increased from 142 in 1999 to 285 in 2011, while the amount of prefectures with 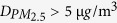 increased from 69 in 1999 to 108 in 2011, and the amount of prefecture with 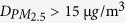 was more than doubled from 12 in 1999 to 25 in 2011. However, the amount of prefectures with 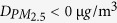 was decreased from 108 in 1999 to 65 in 2011, and the amount of prefectures with 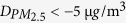 decreased from 13 in 1999 to 6 in 2011(Fig. 2).

**Figure 3:**
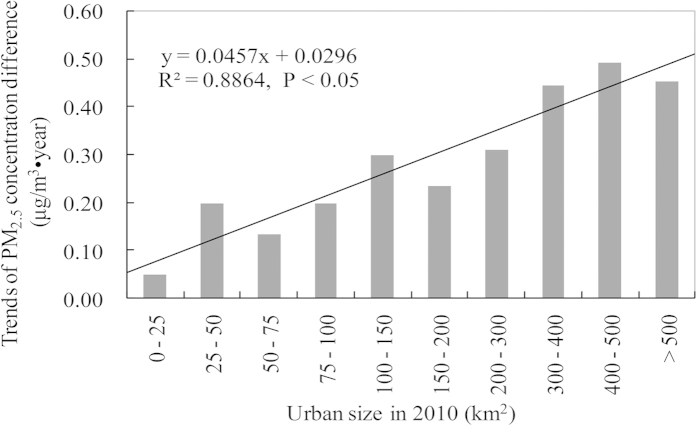
Relationship between urban size in 2010 and trends in PM_2.5_ concentration differences (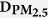) between urban and rural areas in China’s prefectures from 1999 to 2011.

Urban size influenced 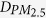, with a significant positive relationship found between the trends of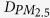 and urban size (Fig. 3; R^2^ = 0.8864, P < 0.05). The trend with more 0.4 μg/m^3^_•_year was obtained at cities with more than 300 km^2^, however, the trend with less than 0.4 μg/m^3^_•_year was obtained at cities with less than 100 km^2^.

**Figure 4:**
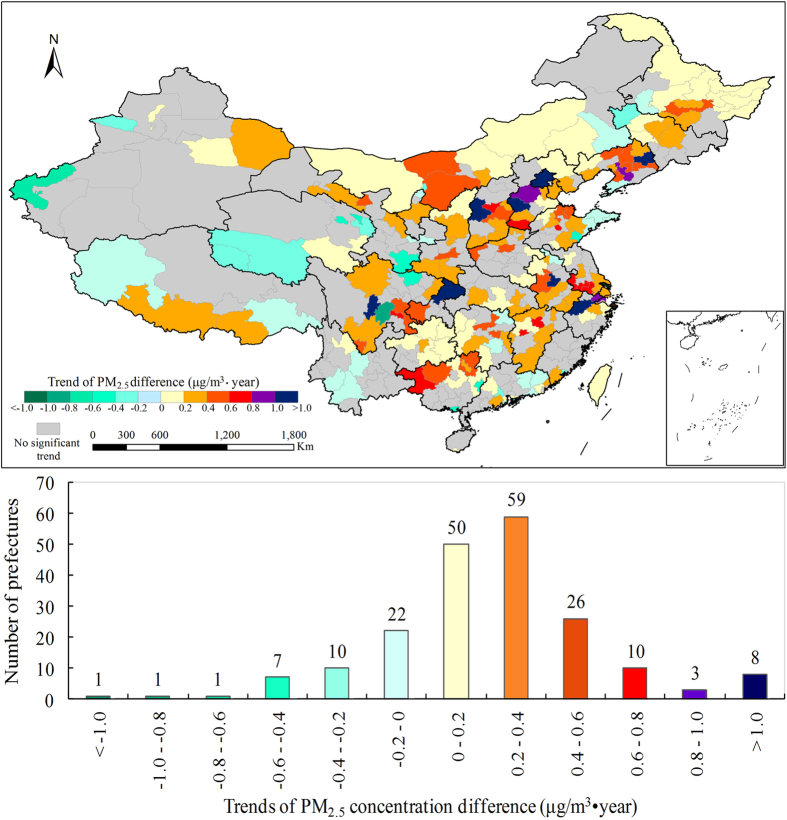
Spatial pattern of trends of PM_2.5_ concentration difference (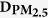) between urban and rural areas from 1999 to 2011. (This figure was created by L. Han in ArcGIS software)

The spatial pattern of 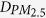 trend at China’s prefectures showed a similar spatial pattern to the 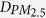 (Fig. 4; Supplementary material 2). Only 42 prefectures, which were mainly located in west and central China, showed significant negative 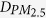 trends. Conversely, 156 prefectures, which were located along two belts from northeast China to the Sichuan province and from Shanghai to the Guangxi province, showed significant positive 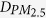 trends. The first belt showed much stronger increasing trend than that in the second belt (Fig. 4).

## Discussion

Urbanization can both positive and negative impact on rapid developing countries. Accelerating urbanization is considered important for economic development in China^13^. China’s central government recently released the National New-type Urbanization Plan that sets the target for urban population fraction at 52.6% in 2012 to reach 60% by 2020^14^. Such rapid growth will drive an increase in economic development and reduction in regional income disparity. However, rapid urbanization also enhances the magnitude of human activities, which contribute to pollution of urban and surrounding environment. Under current strategy, China’s cities would bring much stronger negative impact on the surrounding areas, especially under the expansion of larger cities. Further urbanization in China must consider and establish stricter rules to conserve urban areas and the surrounding environment. Practically, the 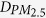 values used in this work provide supplementary criteria to the 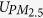/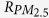 data in order to quantitatively illustrate the influence of urbanization on environment.

Urbanization and its environmental impact experiencing different spatial patterns, suggested various urbanization and environmental protection policies should be considered and taken in different areas of China. China’s current urbanization has mainly occurred in eastern and central China, with less than half the nation’s land supporting more than 90% of the population^15^,^16^. The highest concentration of PM_2.5_ were also observed mainly along the east China plain area^3^,^12^. The 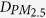 trends obtained in this study showed similar spatial patterns to those reported in previous research^3^, which illustrated strong negative impacts of the urban environment in eastern and central China along two belts from Beijing to Sichuan and from Shanghai to Guangxi. Those patterns indicate different environmental protection policies or actions are required. For instance, under rapid urbanizing, heavy pollution, and strong negative impact of urban areas on the surrounding environment, very strict pollutants emission control and policy should be applied, however, under slow urbanization, light pollution, and no clear impact of urban areas to the surrounding environment, the moderate environmental policies should be adopted.

## Methods

### Study area

Prefecture is the basic administrative unit between province and county, and can be used to demonstrate China’s urban environmental pollution. We therefore took prefectures as the basic study unit to quantify the impact of urban PM_2.5_ concentration on the surrounding areas (Supplementary material 1).

### Fine particulate matter (PM_2.5_) data

The PM_2.5_ concentration used in this research was estimated with an optimal estimation algorithm based on top-of-atmosphere reflectance observed by Moderate Resolution Imaging Spectroradiometer (MODIS) products^9^,^10^. In practice, based on GEOS-Chem chemical transport model simulation, PM_2.5_ concentrations were estimated from the combination of MODIS and Multi-angle Imaging SpectroRadiometer (MISR) AOD with aerosol vertical profiles and scattering properties^9^,^10^. The global PM_2.5_ concentration dataset had a spatial resolution of 10 km as three years moving average from 1999 to 2011^10^. The approach achieved significant agreement (r = 0.81; slope = 0.68) between satellite-derived estimates and ground-based measurements outside North America and Europe was obtained, including many ground measurements in China. It thus provides greater possibility in large regional study of PM_2.5_ concentration’s dynamic^10^. The dataset can be directly download from Atmospheric Composition Analysis Group at Dalhousie University (Website: http://fizz.phys.dal.ca/~atmos/martin/). We used a subset of the global PM_2.5_ concentration dataset that covered China from 1999 to 2011.

### Urban distribution and prefectural boundary

Urban distribution with a spatial resolution of 1 km was used to identify urban from non-urban areas in 2000 and 2010^15^. The prefectural boundary layer with a scale of 1:250,000 was obtained from the National Geomatics Center of China (http://ngcc.sbsm.gov.cn/).

### PM_2.5_ concentration differences

The PM_2.5_ concentration in the urban/non-urban areas (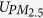/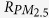) were firstly calculated with based on the urban map in each Chinese prefecture. 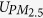/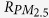
was collected and averaged from the intersection of urban/non-urban areas between 2000 and 2010 to avoid the spatial inconsistency from urban expansion. The differences in PM_2.5_ concentration (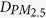) between 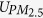 and 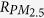 were then obtained for each individual year during 1999–2011 with equation (1)^3^.
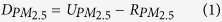
Then the relationship between 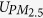 and 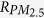 trends was then examined to explain the changes of 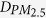. In addition, we compared urban sizes and the 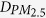 trend to explain the different impacts of urban size on the surrounding areas. Further spatial pattern of 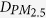 trend, when R > 0.5 and P < 0.05, was finally obtained and analyzed to illustrate geographical “hot-spot” of urban air pollution on surrounding areas.

Current calculation of 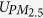/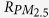 is only based on two years’ (2000 and 2010) urban maps’ intersection which could introduce errors to 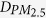 for each year during 2000–2010. In future, when annual urban map available, we suggest to calculate 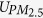/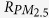 based on each year’s urban cover map to minimize the uncertainty.

## Additional Information

**How to cite this article**: Han, L. *et al.* Increasing impact of urban fine particles (PM_2.5_) on areas surrounding Chinese cities. *Sci. Rep.* **5**, 12467; doi: 10.1038/srep12467 (2015).

## Supplementary Material

Supplementary Information
