# Supplementary material for: Increasing impact of urban fine particles (PM2.5) on areas surrounding Chinese cities
Source: Sci Rep. 2015 Jul 29;5:12467. doi: 10.1038/srep12467 (PMC4518225; doi:10.1038/srep12467)

Supplementary Materials

Increasing impact of urban fine particles ($PM_{2.5}$) on areas surrounding Chinese cities

Lijian Han *,[a], Weiqi Zhou [a], and Weifeng Li [a]

a: State Key Laboratory of Urban and Regional Ecology, Research Center for Eco-Environmental Sciences, Chinese Academy of Sciences, Beijing 100085, China.

***** Corresponding author: world.han@gmail.com (L. Han)

Tel: +86-10-6291-5372   Fax: +86-10-6291-5372

Author Contribution Statement

L. Han (L. Han) contributed to the literature search, study design, data analysis and interpretation, and manuscript writing and revision.

W. Zhou (W. Zhou) and **W. Li** (W. Li) contributed to the manuscript writing and revision.

And all authors declare no competing financial interests

Acknowledgements

This research was a part of Project Spatiotemporal Pattern of Urbanized Regions' Particulate Matter (PMx) and Its Relationship with Landscape Pattern supported by Natural Science Foundation of China (NSFC41301199) and Project Sponsored by the Scientific Research Foundation for the Returned Overseas Chinese Scholars, State Education Ministry. In addition, the research received finical support from the Science and Technology Service Network Initiative Project of the Chinese Academy of Sciences (KFJ-EW-ZY-004), Hundred Talents Program of Chinese Academy of Science, and “135” Key Cultivate Project (YSW2013B04) of Research Center for Eco-Environmental Sciences, Chinese Academy of Sciences.

Supplementary Material 1

Chinese prefectures and urban areas in 2010. (This figure was created by L. Han in ArcGIS software)

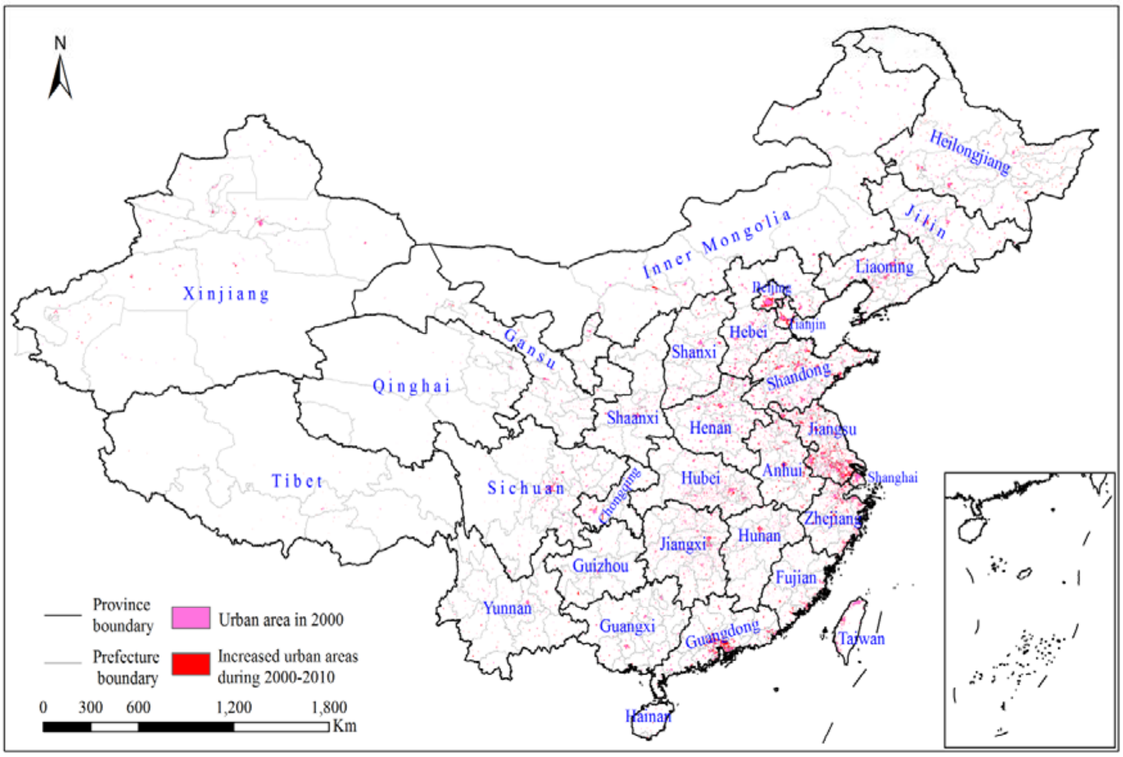

Supplementary Material 2

Spatial pattern of $PM_{2.5}$ concentration difference between urban and rural areas from 1999 to 2011.

(This figure was created by L. Han in ArcGIS software)

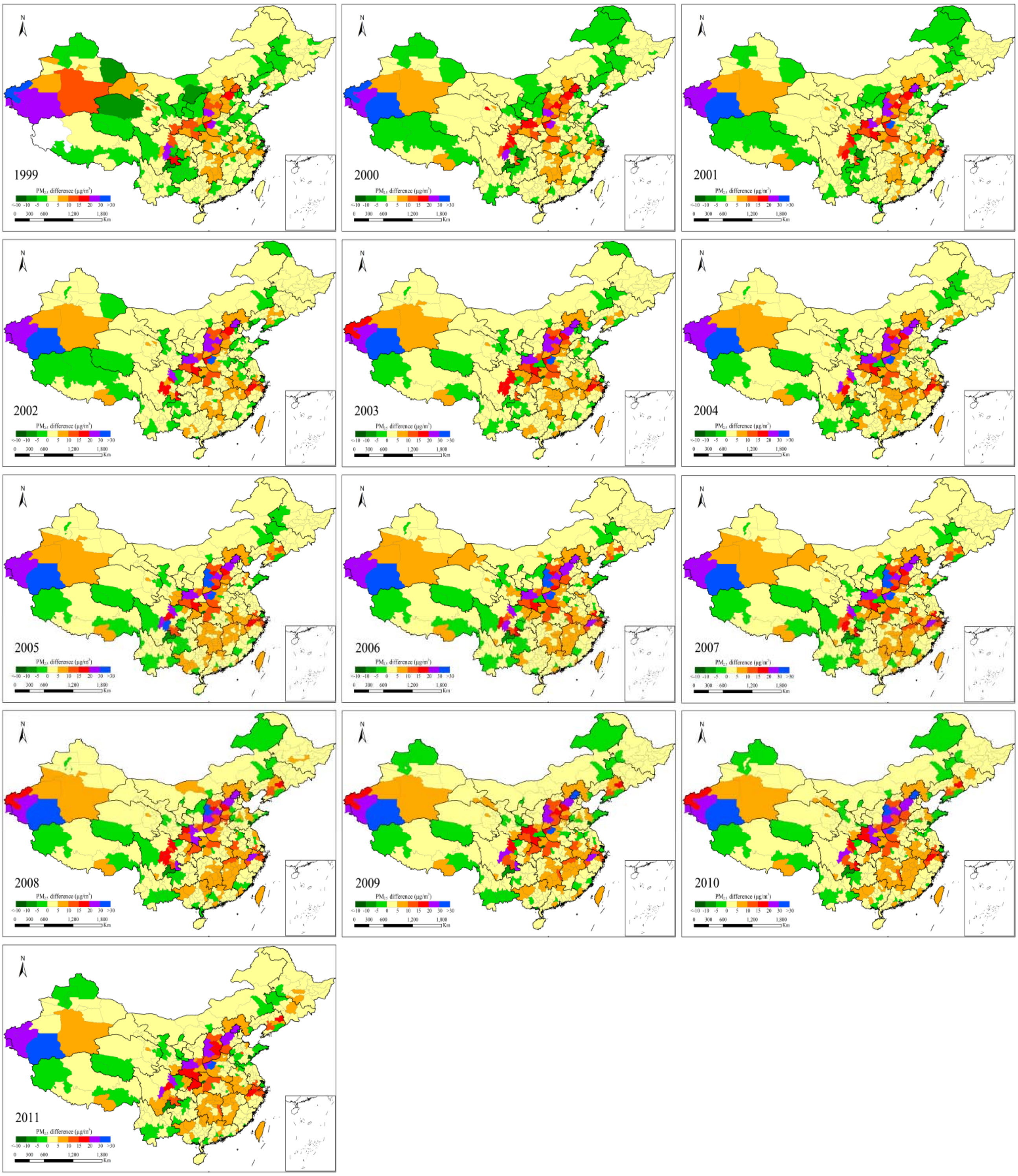

Supplement: Supplementary Information [file srep12467-s1.pdf]
